# A role for the ribosome-associated complex in activation of the IRE1 branch of UPR

**DOI:** 10.1016/j.celrep.2021.109366

**Published:** 2021-07-13

**Authors:** I-Hui Wu, Jae Seok Yoon, Qian Yang, Yi Liu, William Skach, Philip Thomas

In the originally published version of this manuscript, Figure S5E included duplicated fluorescent images. The incorrect version was included due to a technical oversight from the production team.

The production team regrets this error.

## Supplementary Material

1

