# Supplementary material for: A role for the ribosome-associated complex in activation of the IRE1 branch of UPR
Source: Cell Rep. Author manuscript; Available in PMC 2021 Aug 18. (PMC8372851; doi:10.1016/j.celrep.2021.109366)
Supplement: 1 [file NIHMS1724262-supplement-1.pdf]

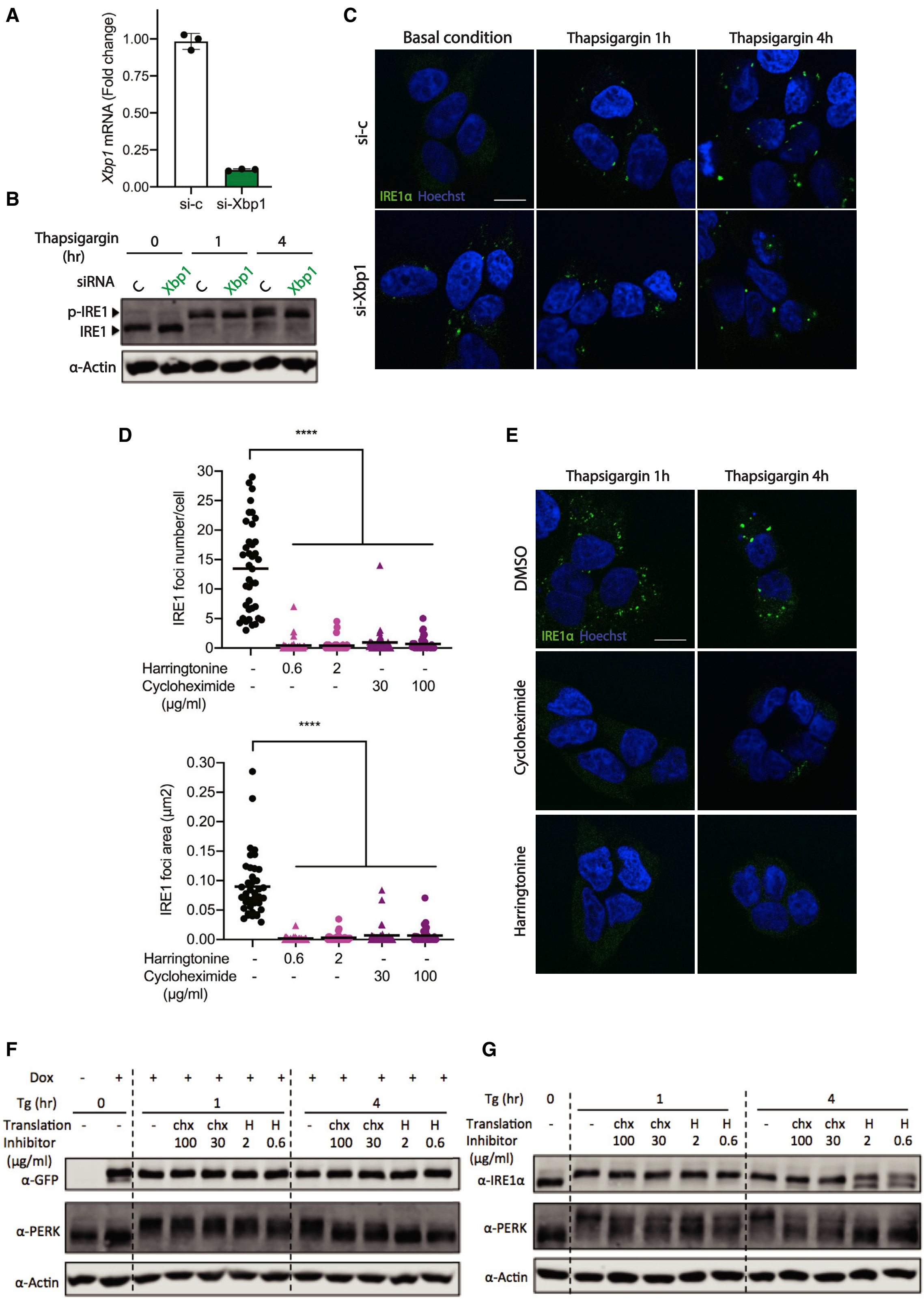

Figure S5. IRE1α clustering is translation dependent, but *Xbp1* independent (corrected)

CellPress
OPEN ACCESS

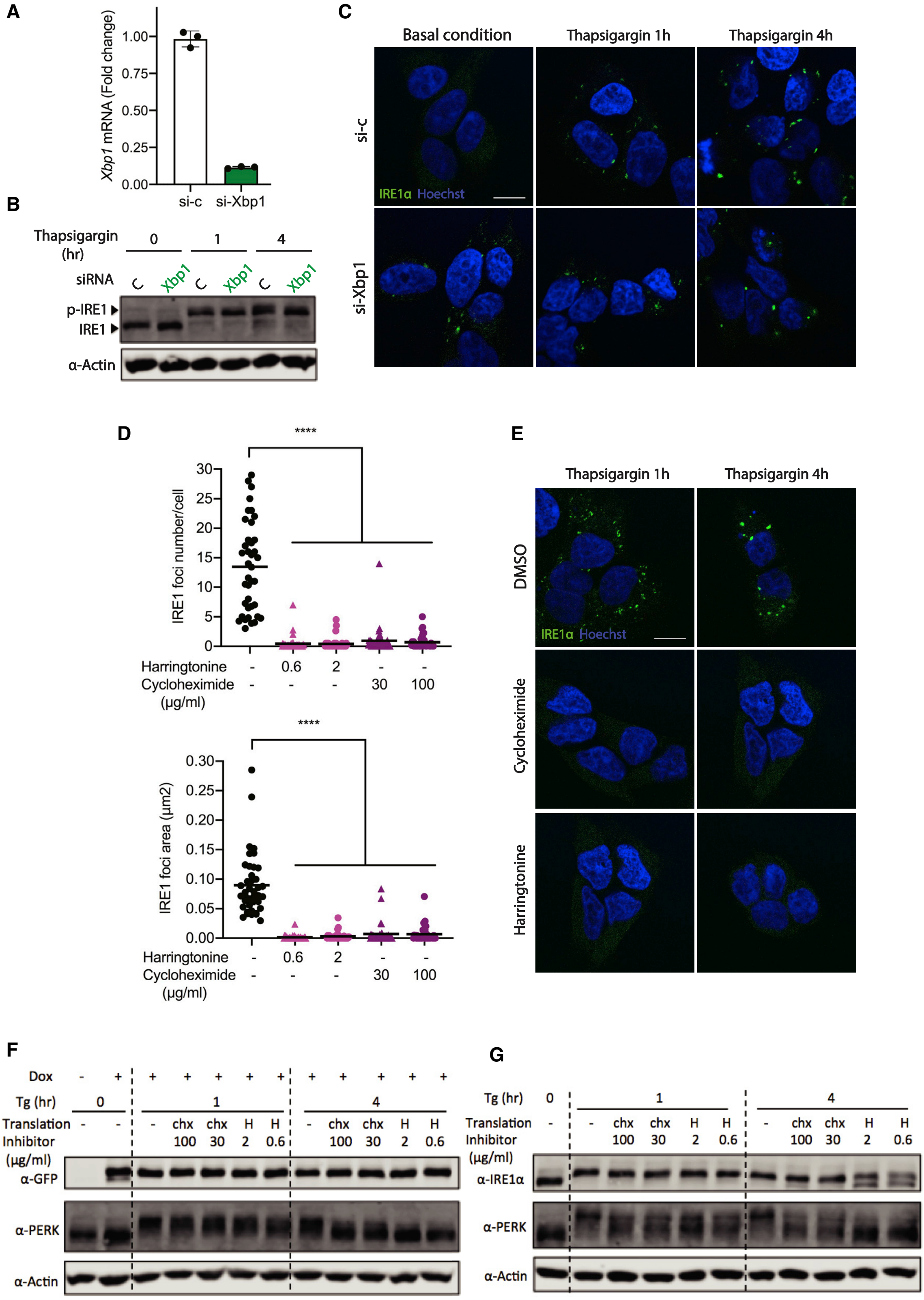

Figure S5. IRE1α clustering is translation dependent, but *Xbp1* independent (original)
